# Psychometric Validation of the Internet Gaming Disorder-20 Test among Ecuadorian Teenagers and Young People

**DOI:** 10.3390/ijerph19095109

**Published:** 2022-04-22

**Authors:** Livia I. Andrade, Marlon Santiago Viñán-Ludeña, Julio Alvarado

**Affiliations:** 1 Department of Psychology, Universidad Técnica Particular de Loja, Loja 110107, Ecuador; jcalvaradox@utpl.edu.ec; 2 Departamento de Ciencias de la Computación e Inteligencia Artificial, ETSI Informática y de Telecomunicación, CITIC-UGR, University of Granada, 18071 Granada, Spain; marsantovi@gmail.com

**Keywords:** IGD-20 test, validation, addiction, students, Ecuador

## Abstract

Excessive internet gaming in high prevalence is a very common problem that has been increasing in recent years, especially in teenagers and university students. However, there is a lack of psychometric evaluation for Internet Gaming Disorder in the Latin American context, particularly in Ecuador. This paper aims to examine the psychometric properties of the Spanish version of the Internet Gaming Disorder test (IGD-20 test) in university and high school students ($n=2931,\;M_{age}\;=\;15.99,$ 57.22% male and 42.51% female). The validation process was performed using one, two, five and six factors taking into account the Spanish, Chinese, Korean, Arabic and Turkish contexts. After checking the models proposed to date, the best fit model was the one with a single factor. Using two samples according to gender (male, female), the invariance has been confirmed with an excellent internal consistency. All, α=0.94; Male, α=0.93; Female, α=0.93). Furthermore, we performed correlation analyses between the IGD-20 Test and socio-demographic variables, and finally, the IGD-20 Test applied to Ecuadorian teenagers and young people demonstrated good psychometric properties.

## 1. Introduction

The Diagnostic and Statistical Manual of Mental Disorders (DSM), according to DSM-IV and DSM-IV-TR, recently included pathological gaming under Impulse Control Disorders, and not classified elsewhere. However, the DSM-5 includes a chapter on behavioral addictions, arguing that these behaviors activate reward systems similar to those activated by drugs and cause some behavioral symptoms similar to those produced by substances, while not related to substance use addiction. However, they possess some characteristics that make them considerable as a potential disorder [1]. In this sense, people with this problem have behaviors that, at first glance, seem normal but are compulsive, which generates loss of freedom from the hook that these activities have (shopping, cell phone use, video games, sex, exercise or even eating). Video game addiction is considered one of the most prevalent behavioral addictions [2]; in this regard, video games are defined as software created for general entertainment and based on the interaction between one or several people and an electronic device that runs it; these electronic devices can be a computer, an arcade machine, a video console, a cell phone, and are known as “platforms” [3].

According to previous research, video games can have some positive effects in some domains such as the following: (i) in education, if they are properly used, they can offer great potential to develop cognitive abilities [4,5,6,7]; in the medical domain, some advances have been achieved in intervention and rehabilitation processes in motor and muscular function and cognitive impairment [8,9,10,11]; in psychology, some studies have shown that videogames improve working memory and verbal capacity. Furthermore, they are useful for entertainment and mental evaluation [12,13,14]. On the other hand, uncontrolled use could cause some negative effects. Video games are characterized by being fun, agile, changing, challenging and easy to use, and they provide rewards and punishments and, therefore, motivate gamers to continue usage, becoming part of people’s daily lives. Video games are popular at all ages; however, teenagers are the ones who use them the most as a regular leisure practice. In addition, there is the possibility of playing with several people at the same time without having to leave home as long as one has access to the internet [15,16,17,18,19]. In this sense, teenagers spend a lot of time on this activity, turning it into a problem with many negative consequences such as: sleep disorders; family and/or social problems [20]; mood disorders; anxiety and personality disorders; and symptoms of prefrontal malfunction and stress [21,22,23]. In addition, other studies have shown that they are related with mental health factors such as depression, anxiety, aggression, expressions of anger, behavioral inhibition and behavioral arousal systems [24,25,26].

The Internet is a technology that has favored the expansion and use of online video games through multiple devices (PC, video game consoles and cell phones) [27], which generates an excessive use and, therefore, a potential problem, incorporating it into categories such as Internet Gaming Disorder (IGD). This disorder is described in Section 3 of DSM-5 and defined as a disorder within behavioural addictions that, in some way, fulfills these nine criteria: (1) concern about online gaming; (2) withdrawal symptoms; (3) tolerance; (4) failure in attempts to control participation in on-line gaming; (5) loss of interest in previous hobbies and entertainment behavior as a result of, and with the exception of internet (online) gaming; (6) excessive and continued use of internet games despite knowledge of the psychosocial problems they cause; (7) misleading of family members, therapists, or others about the amount of online gaming; (8) use of internet games as a form of escape or relief from negative moods; and, (9) risk or lost of significant interpersonal relationships, work, and educational or professional opportunities due to participation in internet gaming. The person must meet at least five of the nine criteria within a 12-month period, although no severity levels are established based on the number of criteria met [1,28].

Similarly, ICD-11, described in Section 6C51, is a gaming disorder characterized by a pattern of persistent or recurrent gaming behaviour (“digital games” or “video games”), which may be internet-based or offline, manifested by: (1) poor control over gaming; (2) increased the priority given to the extent that it takes precedence over other life interests and daily activities; and (3) continuation or intensification of gaming despite the occurrence of negative consequences [29].

It is worth mentioning that there is still not enough research to classify IGD as a disorder in the diagnostic manuals (DSM-5), although, in the ICD-11, it has recently been considered as such, which is why tools were developed to assess these behaviors. An example of these tools is the Internet Gaming Disorder Test IGD-20, developed by [30], which was the first standardized psychometric tool to assess Internet Gaming Disorder (IGD) and was framed based on the nine criteria for Internet Gaming Disorder (IGD) provided by the APA in the DSM-5.

Since 2014, researchers have published different studies covering this topic. In the Table 1, we summarize the IGD-20 validation studies in different countries.

In recent years, there has been an increase in worldwide research on video game addiction; however, in the Ecuadorian population, research about this phenomenon is scarce, considering that in Ecuador, the internet is used by about 57.8% of the population (10.17 million people), and 47% of the worldwide population play video games, according to the reports from We Are Social [35]. Therefore, a standardized and validated instrument that allows us to identify the behavior leading to problematic use of video games is required in the research and diagnosis of clinical disorders. The main aim of this study is to reveal the psychometric properties and validate (in the Ecuadorean context) the Spanish version of the IGD-20 test developed by [26], which highlights the importance of these types of studies since, up to the writing of this document, Ecuador lacks a validated IGD-Test for these kinds of disorders.

## 2. Materials and Methods

### 2.1. Participants and Procedure

The Internet Gaming Disorder IGD-20 was constructed by [30] to assess the problematic use of video games, and it was translated and validated into Spanish by Fuster et al., (2016) [26]. In this psychometric validation, we used the Spanish version. The IGD-20 test includes 20 items that participants rated on a 5-point Likert scale: 1 (“strongly disagree”), 2 (“disagree”), 3 (“neither agree nor disagree”), 4 (“agree”) and 5 (“totally agree”). If the participants obtain a higher total score, it means that they have video game addiction problems. The total score is the sum of the 20 items. The recommended cut-off point for this score is 75. Thus, a totalScore<75 is considered as a person with a non-problematic use, and a totalScore≥75 is considered as an individual with a problematic use of video games.

The study was conducted following the ethical guidelines for research in psychology. First, a research protocol was designed by [36], which helped psychology students of the “Universidad Técnica Particular de Loja”, apply the questionnaire to students in the 10th grade of basic general education, and in the first and second year of secondary school, from public and private educational institutions, in 76 cities in Ecuador, such as Quito, Cuenca, Loja, Guayaquil, etc. Second, young people were recruited from three universities in Loja, including: students from the “Universidad Técnica Particular de Loja”, in initial semesters belonging to different majors: (Psychology, Clinical Psychology, Psychopedagogy, Communication, Architecture, Business Administration, Economics, Chemistry, Biochemistry and Pharmacy, Civil Engineering and Foreign Languages), students from the “Universidad Nacional de Loja” of different majors (i.e., Forestry engineering, Nursing, Environmental Engineering, Systems Engineering, Communication), and finally students from the “Universidad Internacional del Ecuador” (of majors as Law, Engineering in Information and Communication Technology, Architecture and Civil Engineering). The participants were informed that their participation is voluntary and confidential. The directors of the educational centers, the parents and/or legal representatives, the teenagers and university students signed the informed consent and assent form prior to the execution of the objectives of the research. Questionnaires were applied in groups of students using pencil and paper.

Lastly, data was collected in the classrooms in middle schools during 2016 and in universities from Loja-Ecuador during 2020 using non-parametric incidental sampling techniques. Thus, data collection was dependent on availability. The study was approved by the vice-director of research of the “Universidad Técnica Particular de Loja”.

### 2.2. Measures

Together with the IGD-20 Test, we also applied a socio-demographic test as an ad hoc survey designed for data collection related to the main aim of this work. It is composed by 9 short-answer questions which collect data about: age, gender, family type, schooling status (dropout), neighborhood, socioeconomic level, weekly game time, weekends and holidays game time and parental control. The sample is composed of 2931 participants, who were 14- to 28-years old (M=15.99, SD=1.56, female=42.51%). All participants reported that they had played online games before. All the descriptive statistics are described in Table 2.

### 2.3. Data Analysis

Before the validation process, pre-processing or screening the data was needed, fixing or removing incorrect data, unusual cases, duplicate cases and other anomalies. First, we removed missing data; later, the Mahalanobis Distance was performed to identify multivariate outliers within dataset with a threshold value of *p* < 0.001 [37]. Lastly, the Shapiro–Wilk (S–W) test suggested the assumption of normality for the variables involved in this study. The final dataset and its descriptive statistics is shown in Table 2.

In order to validate the IGD-20 test among Ecuadorian students, the Confirmatory Factor Analysis (CFA) was conducted using an open-source R package called Lavaan [38] using maximum likelihood estimation to test structural model. Multisample modeling was conducted according the participants’ gender (male and female) to investigate the IGD-20 Test measurement invariance. The evaluation process was done according to three indicators: (i) Chi-square *p*-value greater than 0.05; (ii) Comparative Fit Index (CFI) greater than 0.90; (iii) Tucker–Lewis fit Index (TlI) greater than 0.90; (iv) Root Mean Square Error of Approximation (RMSEA) less than 0.06 [30,39]; and (v) Standardized Root Mean Square Residual (SRMSR) less than 0.08 [33,39,40].

To examine female and male samples, we follow the recommendations made by Yu et al., (2019) [33]: conducting tests on metric (factor loading invariance) and scalar (measurement intercept) invariances in the model. The overall invariance model was fitted first with an equal factor structure across the two samples, and factor loadings and measurement intercepts to be freely estimated. Then, two restrictive invariance models were fitted to evaluate the invariance across samples. Finally, comparisons were performed with the likelihood ratio test and the change in CFI. To evaluate the invariance, at least two of the following criteria were considered: (i) the $\chi^{2}$ difference is significant with p<0.05; (ii) the change in CFI≥0.01 and (iii) the change in TlI≥0.02.

All these guidelines were used to identify which models best fit the data.

### 2.4. Ethics

This research was done following the ethical guidelines for research in psychology through a protocol developed by Andrade & Ontaneda (2015) [36]. In addition, this study followed the ethical principles included in the Declaration of Helsinki by Mazzanti Di Ruggiero (2011) in its updates, and in the current codes and guidelines such as: consent, personal data protection, confidentiality, non-discrimination, gratuity and the possibility to drop out the study. On the other hand, the students were informed that their participation is voluntary and confidential. Furthermore, the parents and/or legal representatives signed the informed consent, and the students signed the informed assent, prior to the execution of the research objectives. Moreover, the respondents were voluntary, confidential and anonymous.

## 3. Results

First, we tried to replicate the model proposed by Griffiths (2005) [41] composed by six-factor model (i.e., salience, mood modification, tolerance, withdrawal symptoms conflict and relapse). Then we tried a five-factor model proposed by Yu et al., (2019) [33], where they combined the items of the salience and tolerance from the original model. Later, we tried a model composed by 4 and 3 factors; however, this resulted in mis-specification models due to high correlation latent variables.

We tested a two-factor model combining the items from “salience”, “tolerance”, “withdrawal”, “conflict” and “relapse” since these latent variables reflected a higher-order factor of engagement or correlation. This model showed an acceptable fit: $\chi^{2}\left(169\right)=1245.21$, p<0.001; CFI=0.997;TLI=0.997;RMSEA=0.047, 90% CI [0.044–0.049]; SRMR=0.035; and the factor loadings were satisfactorily high (0.618–0.891), except for Items 2 (0.202) and 19 (0.285). However, we tested a model with only one factor, also with an acceptable fit; $\chi^{2}\left(190\right)=1401.553,\;p<0.001;\;CFI=0.997;TLI=0.996;RMSEA=0.05$, 90% CI [0.047–0.052]; SRMR=0.036; and the factor loadings were also high (0.617–0.890), except for items 2 (0.188) and 19 (0.285). In fact, performing Principal Component Analysis (PCA) on the 20-item IGD test revealed the presence of two components with eigenvalues exceeding 1, explaining 49.1% and 5.8% of the variance, respectively. Moreover, it reported a clear break after the first component from the other components. Thus, a one-factor model should be kept in this study. Additionally, the one-factor model was used by Hawi et al., (2017) [31].

### 3.1. Factorial Invariance across Female and Male Samples

Table 3 shows the factor loadings of each item across samples as well as in the whole group.

In the Table 4 the configural invariance showed a good model fit $\chi^{2}\left(418\right)=1800.83$, p<0.001; RMSEA=0.048;CFI=0.997;TLI=0.997;SRMR=0.040. The metric invariance also had a good model fit: $\chi^{2}\left(359\right)=1792.18$, p<0.001; RMSEA=0.052; CFI=0.996; TLI=0.996; SRMR=0.043. The scaled $\Delta \chi^{2}\left(59\right)=8.65,p<0.001$ and ΔCFI=0.001. There was little change in CFI (less than 0.01), indicating that the metric invariance model was maintained across samples. Meanwhile, the scalar invariance model does not change in comparison with the configural model. Thus, our results have shown that both factor loadings and measurement intercepts were equal across male and female samples.

The model in the Table 4 was evaluated to have significant change in goodness of fit compared to the previous model when two of three criteria are met: the $\chi^{2}$ difference was significant (*p* < 0.05), the change in CFI was equal to 0.001 and the change in the TLI was equal to 0.001, and the RSMEA was equal to 0.004, which is in concordance with [42] that it should be less than 0.015.

### 3.2. Reliability and Concurrent Validity

According to Cronbach’s alpha showed in Table 3 the reliability of the IGD-20 test was high as overall as well as for male and female samples.

The relationship between the total IGD-20 test scores and socio-demographic variables (i.e., family type, schooling status (dropout), neighborhood, socioeconomic level, parental control, weekly gameplay, weekends and holidays gameplay, gender and age) were explored to verify concurrent validity. The correlation between video game use during weekdays and IGD-20 is positive (r=0.23, *p* < 0.01). Thus, for teenagers and young people who spend more time in front of video games, the results showed greater symptoms of problematic video game use. Meanwhile the correlation between being a school dropout and IGD-20 showed a moderate inverse relationship (r=−0.56, *p* < 0.01), which indicates that problematic video games use does not have influence over the loss of school years. On the other hand, there were no statistically significant results between IGD-20 and the variables: family type (r=−0.05, *p* < 0.05), neighborhood (r=0.03), socioeconomic level (r=−0.05, *p* < 0.01), weekends and holidays gameplay (r=0.05, *p* < 0.01), parental control (r=0.07,*p* < 0.01), gender (r=−0.05), and age (r=0.11) Table 5.

The cut-off point used is 75 to distinguish gamers with problematic use from gamers with non-problematic use.

Our analysis has shown that the respondents with a problematic use comprised 4.5%, in contrast to 95.5% who reported a non-problematic video games use. According to our results, there exists an important percentage of Ecuadorian teenagers and young people with a problematic use of video games.

## 4. Conclusions

This study revealed the psychometric properties of the Spanish version of the IGD-20 Test applied in Ecuador. First, the reliability analysis showed an excellent internal consistency α=0.94 despite the factor loading of item 2 being quite low. Furthermore, we applied an exploratory factor analysis, with one, two, five, and six factors; the results showed that the best-fitying model was a one-factor model. This result was confirmed using PCA, showing that only one component has 49.1% of the variance. Although other studies work with five and six factors, our model fits the data very well. Video game addiction is considered a mental disease; therefore, the IGD-20 Test is a valid instrument for the use in Ecuador. Thus, this work provides a way to help clinically identify gamers diagnosed with IGD and contributes to the knowledge about IGD for future research in Ecuador and Latin America.

According to the results, our study showed an excellent internal consistency, higher than that of all the works analyzed in Table 1 on the validation of the IDG-20. The invariance test complies with the specifications established by Chen (2007), obtaining better results (DeltaCFI=0.001, DeltaTLI=0.001 and DeltaRSMEA=0.004) than those shown in [33]. The study conducted by [31] presents a single-factor model with alpha=0.91; however, the internal consistency of our study is better. On the other hand, there is a moderate correlation between IGD-20 and weekly playing (*r* = 0.23, *p* < 0.001). A total of 4% of the respondents had symptoms of problematic use, playing between 1 and 4 h per day. Of the students that were school dropouts, 14.8% had symptoms of problematic video game use, while 1.7% of the respondents that were not school dropouts showed symptoms of problematic use; this explains the moderate negative correlation between IGD and being a school dropout (r=−0.56, *p* < 0.01).

This study has some limitations, since it is necessary to work with a more homogeneous dataset. Moreover, answers may be biased based on social desirability as reported in the original and Spanish versions [26,30]. It is important to work with clinical data to test the stability and reliability over the socio-demographic variables considered in our study. Furthermore, work with other disorders, such as internet addiction and the different psychiatric disorders related to video game addiction, may be analyzed in the future. Finally, taking account social media data such as: gamer forums, blogs, social networks platforms in combination with IGD-20 Test may throw interesting results.

## Figures and Tables

**Table 1:** Summary of studies about IGD-20.

Author	Country	Age	Factors	Format(Options)	α	Sample
[30]	Different countries	16–58	Six	Likert(5)	0.88	1003
[26]	Spain	12–58	Six	Likert(5)	0.87	1074
[31]	Saudi Arabia	14–19	Single	Likert(5)	0.91	375
[32]	South Korea	13–55	Six	Likert(5)	0.85	1403
[33]	China	11–28	five	Likert(5)	0.92	1092
[34]	Turkey	10–18	Five	Likert(5)	0.86	1161

**Table 2:** Socio-demographic characteristics of the sample.

	n	%
Gender		
Male	1677	57.22%
Female	1246	42.51%
Other	8	0.27 %
Family Type		
Single parent	685	23.37%
Both parents (nuclear)	1672	57.05%
Extended family	334	11.40%
Others	240	8.18%
School dropout		
Yes	758	25.86%
No	2173	74.14%
Neighborhood		
Urban	2430	82.91%
Suburbs	501	17.09%
Socio-economic level		
Low	758	25.86%
Medium	1319	45.00%
High	854	29.14%
Parental Control		
Yes	912	31.12%
No	2019	68.88%
	**Mean**	**SD**
Age	15.99	1.56
Weekly Gameplay	1.87	1.44
Weekends and holidays Gameplay	1.8	1.65

**Table 3:** Factor loadings of the IGD-20 test and Cronbach’s α.

IGD-20	Male (α=0.93)	Female (α=0.93)	All (α=0.94)
1. I often lose sleep because of long gaming sessions	0.573	0.669	0.617
2. I never play games in order to feel better	0.143	0.257	0.199
3. I have significantly increased the amount of time I play games over past year	0.707	0.761	0.731
4. When I am not gaming I feel more irritable	0.744	0.825	0.781
5. I have lost interest in other hobbies because of my gaming	0.788	0.843	0.812
6. I would like to cut down my gaming time but it’s difficult to do	0.674	0.731	0.699
7. I usually think about my next gaming session when I am not playing	0.784	0.868	0.821
8. I play games to help me cope with any bad feelings I might have	0.757	0.795	0.773
9. I need to spend increasing amounts of time engaged in playing games	0.881	0.903	0.890
10. I feel sad if I am not able to play games	0.837	0.885	0.858
11. I have lied to my family members because the amount of gaming I do	0.809	0.854	0.829
12. I do not think I could stop gaming	0.799	0.848	0.819
13. I think gaming has become the most time consuming activity in my life	0.833	0.863	0.845
14. I play games to forget about whatever’s bothering me	0.775	0.803	0.787
15. I often think that a whole day is not enough to do everything I need to do in-game	0.817	0.892	0.850
16. I tend to get anxious if I can’t play games for any reason	0.829	0.879	0.851
17. I think my gaming has jeopardised the relationship with my partner	0.693	0.771	0.728
18. I often try to play games less but find I cannot	0.780	0.852	0.812
19. I know my main daily activity (i.e., occupation, education, homemaker, etc.) has not been negatively affected by my gaming	0.314	0.246	0.285
20. I believe my gaming is negatively impacting on important areas of my life	0.649	0.699	0.672

**Table 4:** Tests of invariance of the IGD-20 Test.

Model	Model Fit						Model Comparison					
	$\chi^{2}$	df	CFI	TLI	RMSEA [90% CI]	SRMR		$\Delta \chi^{2}$	Δdf	ΔCFI	ΔTLI	ΔRSMEA
Configural	1800.83	418	0.997	0.997	0.048 [0.045–0.050]	0.040						
Metric	1792.18	359	0.996	0.996	0.052 [0.050–0.055]	0.043		8.65	59	0.001	0.001	0.004
Scalar	1800.83	418	0.997	0.997	0.048 [0.045–0.050]	0.040		8.65	59	0.001	0.001	0.004

Note: CFI—comparative fit index; TLI—Tucker–Lewis index; RMSEA—root mean square error of approximation; SRMR—standardized root mean square residual; CI—confidence interval; IGD-20 Test—20-item Internet Gaming Disorder Test.

**Table 5:** Concurrent and convergent validity of the IGD-20 Test.

	1	2	3	4	5	6	7	8	9	10
1. IGD-20	–	−0.05 *	−0.56 **	0.03	−0.05 **	0.07 **	0.23 **	0.05 **	−0.06	0.12
2. Family type		–	0.11 **	0.00	0.03	−0.01 **	0.03	−0.00	0.05	−0.02 *
3. School dropout			–	0.05 **	0.03 **	−0.24 **	−0.16 **	0.21 **	0.16 **	−0.51 **
4. Neighborhood				–	−0.18 **	−0.04	−0.07 **	−0.01	0.07	−0.02
5. Socioeconomic level					–	−0.07 **	0.11 **	0.05 **	0.03 **	−0.05 **
6. Parental control						–	−0.03 **	−0.32 **	−0.24 **	0.14 **
7. Weekly gameplay							–	−0.12 **	0.01 **	0.14 **
8. Weekends and Holidays gameplay								–	0.29 **	−0.10 *
9. Gender									–	−0.12 **
10. Age										–
Mean	40.33	1.99	1.72	1.13	2.03	0.63	1.87	1.80	1.57	15.99
SD	16.23	0.82	0.47	0.39	0.34	0.74	1.44	1.65	0.5	1.55

Note: * (*p* < 0.05); ** (*p* < 0.01).

## Data Availability

The data presented in this study are available on request from the corresponding author.
